# Saccadic Eye Movements in Elderly Depressed Patients With Suicidal Behaviors: An Exploratory Eye-Tracking Study

**DOI:** 10.3389/fpsyg.2021.712347

**Published:** 2021-11-11

**Authors:** Yoan Barsznica, Nicolas Noiret, Bérénice Lambert, Julie Monnin, Claire De Pinho, Julia Hickel, Caroline Masse, Stephane Richard-Devantoy, Cynthia Morgny, Djamila Bennabi, Emmanuel Haffen, Eric Laurent, Pierre Vandel, Gilles Chopard

**Affiliations:** ^1^Department of Clinical Psychiatry, Besançon University Hospital, Besançon, France; ^2^Department of Neurology, Besançon University Hospital, Besançon, France; ^3^Laboratory of Neurosciences and Cognitive Psychology, University of Bourgogne Franche-Comté, Besançon, France; ^4^Memory Center of Research and Resources (CMRR), Besançon University Hospital, Besançon, France; ^5^UMR CNRS 7295 “Research Centre on Cognition and Learning,” University of Poitiers, Poitiers, France; ^6^Regional Health Observatory, Bourgogne-Franche-Comté, France; ^7^Department of Old Age Psychiatry, Association Hospitalière de Bourgogne Franche-Comté Hospital, Bavilliers, France; ^8^McGill Group for Suicide Studies, Douglas Mental Health University Institute, Department of Psychiatry, McGill University, Montreal, QC, Canada

**Keywords:** depression, elderly suicide, executive function, eye movement analysis, eye tracking

## Abstract

Suicidal behaviors (SBs) are often associated with impaired performance on neuropsychological executive functioning (EF) measures that encourage the development of more specific and reliable tools. Recent evidence could suggest that saccadic movement using eye tracking can provide reliable information on EF in depressive elderly. The aim of this study was to describe oculomotor performances in elderly depressed patients with SB. To achieve this aim, we compared saccadic eye movement (SEM) performances in elderly depressed patients (*N* = 24) with SB and with no SB in prosaccade (PS) and antisaccade (AS) tasks under the gap, step, and overlap conditions. All participants also underwent a complete neuropsychological battery. Performances were impaired in patients with SB who exhibited less corrected AS errors and longer time to correct them than patients with no SB. Moreover, both groups had a similar performance for PS latencies and correct AS. These preliminary results suggested higher cognitive inflexibility in suicidal patients compared to non-suicidal. This inflexibility may explain the difficulty of the depressed elderly in generating solutions to the resurgence of suicidal ideation (SI) to respond adequately to stressful environments. The assessment of eye movement parameters in depressed elderly patients may be a first step in identifying high-risk patients for suicide.

## Introduction

Around one million people die by suicide and ten million people make a suicide attempt (SA) each year worldwide (World Health Organization, 2014). Suicide is therefore a major public health problem, especially among the elderly population (Shah et al., 2016). The ratio between SA and completed suicide has been estimated to be 4:1 in the elderly versus 200:1 in young adults (De Leo et al., 2001; Conwell and Thompson, 2008). Additionally, suicide is often associated with unipolar depression among the elderly (Alexopoulos et al., 1999; Szanto et al., 2001). Thus, age-related suicide vulnerability associated with depression requires great vigilance (Bazin, 2004) and emphasizes the need to identify high-risk patients.

Age-related suicide vulnerability could be explained by cognitive and emotional inability to respond adequately to stressful environmental factors (Richard-Devantoy et al., 2012). In accordance with this proposal, executive function (EF) seems to play a crucial role in the suicide vulnerability in aging people in that it refers to abilities required to facilitate adaptation to novel and/or complex situations (Jurado and Rosselli, 2007). Elderly depressed patients with a history of SA or suicidal ideation (SI) are found to have poorer executive performance than non-suicidal depressed and healthy elderly especially on tasks that rely on inhibitory control and cognitive flexibility (King et al., 2000; Marzuk et al., 2005; Westheide et al., 2008; Mcgirr et al., 2012; Richard-Devantoy et al., 2012, 2015, 2016).

From a clinical perspective, the neuropsychological tests measuring EFs, such as the Trail Making Test (TMT) B (Richard-Devantoy et al., 2012) or the Stroop test (Stroop, 1935; Meulemans, 2008), might be used as a potential predictive indicator of the risk of suicidal behavior (SB). However, the neuropsychological tests do not provide a pure measure of EF and include other non-executive processes which make validation difficult (Miyake et al., 2000). Furthermore, it is well established that an isolated low score may have little relevance in clinical evaluation (Iverson et al., 2011). Therefore, it is still too early to use neuropsychological tests as predictors of SB (Richard-Devantoy et al., 2013). This should encourage the development of more specific and reliable tools.

Another approach for assessing executive impairment is based on oculomotor measurements. Eye-tracking tasks allow more detailed analysis and limiting the measurement bias of neuropsychological tests (for example, launching the task, timing, language, or motor difficulties). They are simple, short, and easily understandable for patients. Among the different tasks used in eye tracking are the prosaccade (PS) and antisaccade (AS) tasks (Leigh and Kennard, 2004; Hutton and Ettinger, 2006). In the PS task, participants are typically instructed to look from a central fixation dot toward a sudden onset peripheral target dot as quickly as possible. Although PS latency usually serves as a measure of processing speed (Carvalho et al., 2014; Noiret et al., 2017), it is also used to investigate the process of disengagement of attention by introducing gap periods between the disappearance of the central fixation dot and the appearance of the target dot (known as gap and overlap saccadic paradigms) (Saslow, 1967; Fischer and Boch, 1983; Kalesnykas and Hallett, 1987; Braun and Breitmeyer, 1988; Fischer et al., 1993). In the gap condition, attention is not focused on the central dot when the target appears, resulting in faster saccadic engagement on the target, and so faster saccadic latency than overlap conditions (Pratt et al., 2006). In AS task, participants are instructed to refrain from looking at the peripheral target dot and direct their gaze in the opposite direction. The AS task is used to investigate the ability to inhibit saccades toward the stimulus and to correct potential saccade errors (i.e., saccade toward the peripheral target dot). In this sense, AS measures (e.g., latency, proportions of AS correct and corrected, and the time to correct AS errors) may reliably inform on EF.

Although many studies have used PS and AS tasks in several psychiatric and neurological conditions (Everling and Fischer, 1998; Hutton and Ettinger, 2006; Armstrong and Olatunji, 2012; Carvalho et al., 2014; Levy et al., 2018), few have examined saccadic eye movement (SEM) performances in normal aging or in elderly depression. Noiret et al. (2017) reported that latencies, time to correct AS errors, and proportion of uncorrected AS increased with aging. Elderly depressed patients were found to have longer latency in PS and AS tasks and a higher proportion of uncorrected saccades in AS task (Carvalho et al., 2014).

However, to our knowledge, no information is available concerning oculomotor measurement in elderly depressed patients with SB (Barsznica et al., 2019). The main objective of our research was to describe oculomotor performances in elderly depressed patients with SB. In this exploratory study, we expected these patients would have lower performances in these SEM tasks compared to elderly depressed patients without SB because of poorer EF that was previously reported compared to other populations (King et al., 2000; Marzuk et al., 2005; Westheide et al., 2008; Mcgirr et al., 2012; Richard-Devantoy et al., 2012, 2015). The use of standard overlap and gap conditions is to measure selective attention (Saslow, 1967; Fischer and Boch, 1983; Kalesnykas and Hallett, 1987; Braun and Breitmeyer, 1988; Fischer et al., 1993).

## Materials and Methods

### Population

Thirty-two inpatients aged from 65 to 86 years were recruited in Nord Franche-Comté Hospital, Bavilliers, France. All patients were interviewed by a trained psychiatrist and presented a current major depressive episode according to diagnostic and statistical manual of mental disorders (DSM)-5 criteria (American Psychiatric Association, 2013) and a Montgomery–Åsberg Depression Rating Scale score >20 (MADRS) (Montgomery and Asberg, 1979). The patient with no SB group (*n* = 12) did not present the personal history of SA or recurring SI, according to the Columbia Suicide Severity Rating Scale (CSSRS; Posner et al., 2008). The patient with SB group (*n* = 12) presented a recent history of SA or recurring SI according to the CSSRS. Any patient with a previous medical history of a neurological disease (i.e., head trauma, stroke, dementia, Parkinson’s disease, epilepsy, or brain tumor) or a psychiatric disorder (i.e., schizophrenia, bipolar disorder, and addictive behaviors other than smoking or borderline personality) was not included. All patients had a normal or corrected-to-normal vision and reported no visual disorders.

All patients were taking antidepressant medications at the time of testing. Informed written consent was obtained from all participants prior to enrollment. The research was approved by the Committee for the Protection of Persons and was conducted in accordance with the Declaration of Helsinki as revised in 1989.

### Neuropsychological Assessment

All patients underwent a neuropsychological assessment protocol focused on the assessment of attention/processing speed, EFs, and verbal episodic memory. General cognitive function was assessed by Mini-Mental State Examination (MMSE; Folstein et al., 1975; Kalafat et al., 2003). This battery of tests was designed to ensure that no participant had cognitive impairments associated with dementia.

#### Assessment of Attention and Processing Speed

The TMT, part A (Reitan, 1958; Godefroy, 2008): Participants are required to connect with lines 25 circles numbered from 1 to 25 as quickly as possible.

The Stroop color reading (Stroop, 1935; Meulemans, 2008): Colored rectangles are presented, and participants have to name the colors.

The Stroop word reading (Stroop, 1935; Meulemans, 2008): Participants have to read aloud color names printed in black ink.

The digit span forward task (Wechsler and Naglieri, 2009): Participants are read aloud a sequence of numbers and recall the numbers in the same order.

#### Assessment of Executive Functions

The TMT, part B (Reitan, 1958; Godefroy, 2008): Participants are required to connect numbers and letters alternatively as quickly as possible. The TMT part B–part A was also calculated as a measure of cognitive flexibility independent of processing speed.

The Phonemic Verbal Fluency (PVF; Godefroy, 2008): Participants are asked to generate as many words as possible beginning with the letter “P,” lasting 2 min.

The Semantic Verbal Fluency (SVF; Godefroy, 2008): Participants are asked to generate as many words as possible belonging to the category “animals,” lasting 2 min.

The Alternate Verbal Fluency (AVF; Iudicello et al., 2008): Participants have to continuously alternate words beginning with the letter “P” and words belonging to the category “Animals,” lasting 2 min. A shifting index was computed according to the following formula: total words generated in the AVF subtest/[(PVF + SVF score)/2]. This index was used to assess the shifting cost a participant pays passing from performing the single fluency subtests to performing the AVF subtest (Costa et al., 2014).

The Stroop color interference (C/W, Stroop, 1935; Meulemans, 2008): Participants have to name the ink color of a printed word that spells the name of a different color. A Stroop difference score was computed (i.e., C/W – Stroop color read) as a measure of response inhibition independent of processing speed (Meulemans, 2008).

The digit span backward task (Wechsler and Naglieri, 2009): Participants are read aloud a sequence of numbers and recall the numbers in reverse order. A digit difference score was calculated (i.e., digit span forward – digit span backward) as a measure of data manipulation independent of storage information (Wager and Smith, 2003; Ruscheweyh et al., 2013).

The Frontal Assessment Battery (FAB; Dubois et al., 2000): This battery consists of six subtests exploring the following: conceptualization, mental flexibility, motor programming, sensitivity to interference, inhibitory control, and environmental autonomy. In addition to the global score, we also used the Go-No Go task subscore that explores the domain of inhibitory control.

#### Assessment of Verbal Episodic Memory

The Free and Cued Selective Reminding Test (FCSRT; Grober and Buschke, 1987; Van der Linden et al., 2004): This test assesses the ability to learn a 16 written word list that refers to 16 semantic categories. This test provided an immediate cued recall score (i.e., encoding phase), a total free recall score (i.e., participants are asked to retrieve the words spontaneously), and a total recall score, which was the sum of free and cued recall (i.e., participants are asked to retrieve the words with the help of a semantic cue). The total number of intrusions (i.e., words absent from the list and falsely recalled) was also recorded.

### Apparatus

Saccadic eye movements were recorded using a remote eye-tracking device at a frequency of 250 Hz, an accuracy of 0.4°, and a spatial resolution of 0.03° (RED 500, SMI^®^, Teltow, Germany). We used DELL E6530 Laptop with an Intel Core i7 processor and a 22-inch display screen with a resolution of 1920 × 1080 pixels and a refresh rate at 60 Hz.

### Saccadic Eye Movement Tasks

#### Prosaccade Task

**FIGURE 1:**
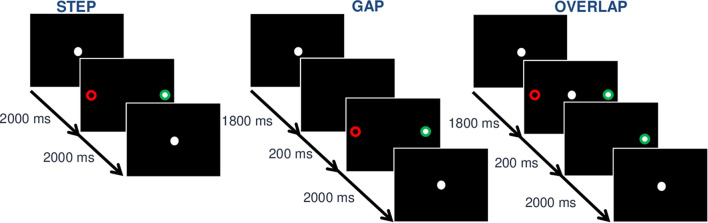
Illustration of the gap, step, and overlap conditions in a paradigm of prosaccade (PS) and antisaccade (AS). The ∘ represents the point displayed, 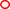 the optimal fixation in the AS task, and 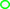 the optimal fixation in PS task. In the step condition (left), the central dot disappears simultaneously with the target dot appearance. In the step condition (left), the central dot simultaneously disappears when the target appears. In the gap condition (middle), the central dot disappears 200 ms before the target dot appears. In the overlap condition (right), the central dot disappears 200 ms after the target dot appears.

Each trial started with a central white fixation point (0.5° of visual angle) on a black background. After 2,000 ms, a white target-point (0.5° of visual angle) appeared for 2,000 ms. Then a new central fixation-point appeared to signal the start of the next trial. In the step condition, the central dot disappears simultaneously with the target dot appearance. In the gap condition, the central dot disappears 200 ms before the target dot appears (i.e., “gap” condition). In the overlap condition, the central dot disappears 200 ms after the target dot appears (Figure 1). Every target-point was displayed with an eccentricity of ±8° or ±16° of visual angle in the horizontal plane. Participants were instructed to keep their gaze on the central fixation-point until the peripheral target point appeared and at this time, they had to look at the target point as accurately and quickly as possible.

#### Antisaccade Task

The AS task was similar to the PS task except for the instructions given to the participants (see Figure 1). Participants were instructed to keep their gaze on the central fixation-point until the peripheral target-point appeared. After the target-point onset, they had to direct their gaze in the opposite direction to the target-point as quickly and accurately as possible.

### Procedure

The experiment was divided into two sessions. In the first session, patients underwent a neuropsychological assessment. In the second session, patients performed the SEM tasks. Neuropsychological tests and SEM tasks were performed 1 week apart. At the beginning of the second session, the patient was seated in a quiet room, 60 cm in front of the monitor, and the eye-tracking system. Each participant completed two blocks of 48 trials. Block orders were counterbalanced between patients (i.e., PS-AS or AS-PS) with a 5-min break and a 5-point calibration between each block. The three conditions were randomly distributed in each block (i.e., 16 steps, 16 gaps, and 16 overlaps). Five practice trials for each block were performed to ensure that participants understood instructions.

### Data Reduction and Analysis

Saccade onset and offset were defined by a fixed velocity threshold of 30°/s. The direction of a saccade was determined by the eye position difference between the start and the end of the saccade. Trials containing anticipated saccades (less than 80 ms) or delayed saccades (more than 800 ms) at the target onset were excluded from the analysis. Trials were also excluded when the eye tracker failed to record the eye coordinates (e.g., eye blink, loss of pupil, or corneal reflection).

Saccades directed toward the targets in the PS task and in the opposite direction in AS task were defined as correct saccades. Saccades directed toward the targets in AS task were defined as AS errors. PS errors were not analyzed because their number was too low for an informative statistical analysis. In AS task, when a subsequent saccade goes in the opposite direction after an AS error, the former was classified as a corrected AS error. Finally, we calculated a gap-effect index [i.e., gap–step ratios: (gap − step)/(gap + step)] and overlap–effect ratios [i.e., overlap–step ratios: (overlap − step)/(overlap + step)] for each SEM latency just cited above.

As the Shapiro–Wilk’s test did not show non-normality distributions and the Levene’s test did not show heterogeneity of the variances for all SEM parameters, we decided to use parametric ANOVA. Patients (patients with SB vs. patients with no SB) were the between-subject factor and conditions (gap, step, and overlap) the within-subject factor. When the assumption of sphericity was violated, we used the Greenhouse–Geisser correction.

## Results

### Demographic, Clinical, and Neuropsychological Variables

As indicated by Table 1, participant groups did not differ significantly on demographic variables, such as male/female ratio, age, and years of education. There was no statistically significant difference according to the severity level of depression. There were no statistically significant differences in cognition performance between the two participant groups.

**TABLE 1:** Demographic, clinical, and neuropsychological characteristics of the study sample.

Variables	SB patients	no SB patients	*t*, *U* or *χ^2^*	*p*	Cohen’s *d*
Sex (male/female)	5/7	3/9	0.75^b^	0.39	
Age	76.25 (6.70)	73.00 (6.72)	0.67	0.51	0.27
Education (years)	7.67 (3.31)	8.42 (3.82)	68.50^a^	0.84	0.21
MADRS	28.50 (5.13)	25.00 (4.31)	1.81	0.08	0.74
MMSE	24.75 (2.70)	24.92 (2.68)	–0.15	0.88	0.06
FCSRT Immediate recall	13.00 (2.80)	14.92 (1.44)	42.50^a^	0.08	0.86
FCSRT Free recall	19.83 (7.63)	26.00 (7.43)	–2.01	0.06	0.82
FCSRT Total recall	41.58 (5.87)	44.42 (4.62)	43.00^a^	0.10	0.54
FCSRT Intrusions	1.83 (3.13)	0.75 (1.48)	58.00^a^	0.38	0.44
SVF	16.42 (7.82)	16.00 (6.51)	0.14	0.89	0.06
PVF	13.33 (4.10)	13.25 (5.82)	0.04	0.97	0.02
AVF	12.3 (8.94)	8.83 (5.15)	1.18	0.25	0.48
Shifting Index	1.11 (1.39)	0.62 (0.36)	69	0.88	0.49
TMT A (s)	66.33 (26.33)	69.09 (36.95)	61.00^a^	0.78	0.09
TMT B (s)	257 (47.9)	236 (59.1)	0.99	0.33	0.40
TMT B-A (s)	202 (43.2)	175 (45.4)	1.47	0.16	0.60
Stroop C	87.42 (25.39)	79.00 (12.07)	48.50^a^	0.29	0.42
Stroop W	60.17 (15.41)	64.73 (25.23)	65.50^a^	1.00	0.22
Stroop C/W	206 (72.3)	182 (35.7)	0.94	0.36	0.38
Stroop C/W – C	118 (55.7)	102 (42.0)	0.83	0.41	0.34
Digit span forward	4.75 (0.87)	4.42 (0.79)	55.50^a^	0.28	0.40
Digit span backward	3.75 (0.97)	3.42 (0.67)	58.00^a^	0.38	0.40
Digit difference score	1.00 (0.74)	1.00 (0.95)			
FAB total score	13.75 (2.14)	14.67 (2.50)	–0.97	0.35	0.39
FAB (Go-No Go)	1.67 (1.07)	2.17 (1.27)	–1.04	0.31	0.43

*If the data did not comply with the *t*-test parameters (heterogeneity or normality), we used Mann–Whitney *U*-test as a non-parametric statistical test. For frequency data, we used the chi-square test (*χ^2^*). “a” exponent: Mann–Whitney test, “b” exponent: the chi-square test. SB, suicidal behaviors; MADRS, Montgomery-Åsberg Depression Rating Scale; MMSE, Mini-Mental State Examination; FCSRT, Free and Cued Selective Reminding Test; SVF, Semantic Verbal Fluency; PVF, Phonemic Verbal Fluency; AVF, Alternate Verbal Fluency test; TMT, Trail Making Test; Stroop C, Stroop color reading; Stroop W, Stroop Word reading; Stroop C/W, The Stroop Color interference; FAB, Frontal Assessment Battery.*

### Saccadic Eye Movement Variables

#### Prosaccade Task

**FIGURE 2:**
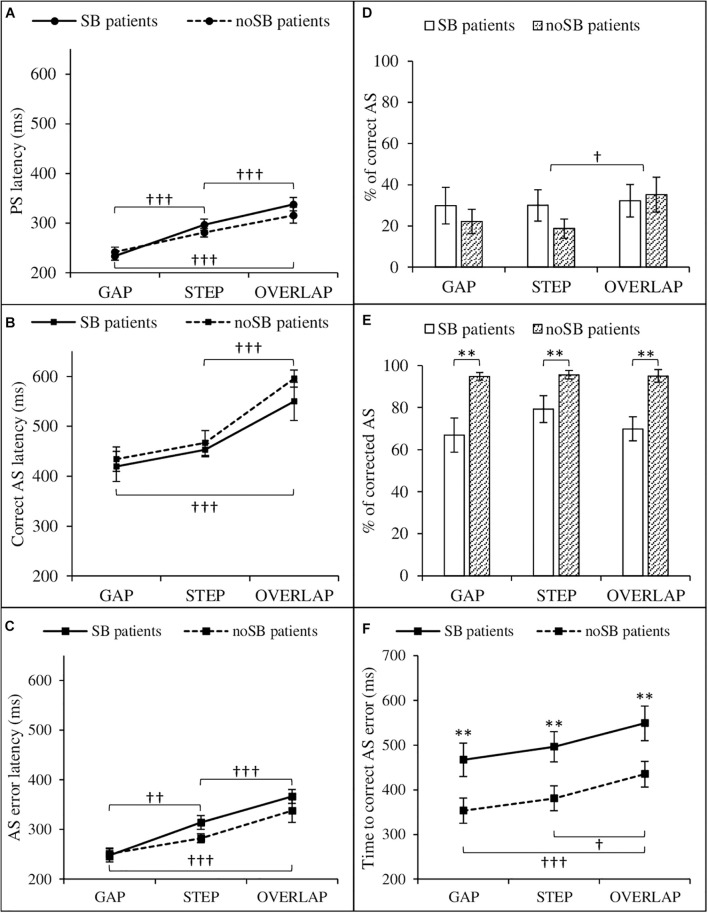
Saccadic eye movement parameters as a function of condition (gap, step, and overlap) and patient group (SB patients and no SB patients). Left: prosaccades **(A)**, correct antisaccades **(B)**, and antisaccade errors **(C)** latencies. Right: proportion (i.e., percentage of the total number of saccades) of correct antisaccades **(D)**, proportion of corrected antisaccades among antisaccade errors **(E)**, time to correct antisaccade errors **(F)**. AS, antisaccade; no SB, no suicidal behaviors; PS, prosaccade; SB, suicidal behaviors. Error bars: ±1 standard errors. Significant differences between condition are represented by “^†^” (^†^*p* < 0.05; ^†^^†^*p* < 0.01; ^†^^†^^†^*p* < 0.001). Significant differences between patients with SB and patients with no SB are represented by “^∗^” (^∗∗^*p* < 0.01).

Analysis of variance on PS latency revealed only a main effect of conditions, *F*(2,44) = 66.09, *p* < 0.001, η*^2^_*p*_* = 0.75. Saccades were faster in gap condition (*M* = 237.62, SD = 30.01) than in step (*M* = 288.45, SD = 35.19, *p* < 0.001) and overlap conditions (*M* = 326.62, SD = 50.97, *p* < 0.001), and they were faster in step than overlap conditions (*p* < 0.001). The main effect of patients [*F*(1,22) = 0.54, *p* = 0.47, η*^2^_*P*_* = 0.02] and the patients × conditions interaction [*F*(2,44) = 2.13, *p* = 0.13, η*^2^_*P*_* = 0.09] were not statistically significant (Figure 2).

As regards the gap–step and the overlap–step ratios, a main effect of conditions was found [*F*(1,22) = 129.63, *p* < 0.001, η*^2^_*p*_* = 0.86]. Gap–step ratio (*M* = − 0.10, SD = 0.06) was lower than the overlap–step ratio (*M* = 0.06, SD = 0.06). Gap–step ratio and overlap–step ratio were statistically different from zero [*t*(23) = −7.71, *p* < 0.001, *t*(23) = 4.47, *p* < 0.001, respectively]. The main effect of patients [*F*(1,22) = 0.52, *p* = 0.48, η*^2^_*P*_* = 0.02] as well as patients × conditions were not statistically significant (see Figure 2).

#### Antisaccade Task

##### Correct Antisaccade

We found only a main effect of conditions on correct AS latency, *F*(2,44) = 28.49, *p* < 0.001, η*^2^_*P*_* = 0.56. Saccades were longer in overlap condition (*M* = 581.24, SD = 100.86) than in step (*M* = 459.55, SD = 67.93, *p* < 0.001) and gap conditions (*M* = 426.72, SD = 93.88, *p* < 0.001). There was no statistical difference between step and gap conditions (*p* = 0.41). The main effect of patients [*F*(1,22) = 1.35, *p* = 0.26, η*^2^_*P*_* = 0.06] and the patients × conditions interaction [*F*(2,44) = 0.84, *p* = 0.44, η*^2^_*P*_* = 0.04] were not statistically significant.

The gap–step and the overlap–step ratios, a main effect of conditions was found [*F*(1,22) = 41.25, *p* < 0.001, η*^2^_*p*_* = 0.65]. Gap–step ratio was (*M* = −0.04, SD = 0.12) lower than the overlap–step ratio (*M* = 0.11, SD = 0.10). Overlap–step ratio was statistically different from zero [*t*(23) = 5.59, *p* < 0.001], contrary to gap–step ratio [*t*(23) = −1.89, *p* = 0.07]. The main effect of patients [*F*(1,22) = 0.89, *p* = 0.35, η*^2^_*P*_* = 0.04] and patients × conditions interaction [*F*(1,22) = 0.67, *p* = 0.42, η*^2^_*P*_* = 0.03] were not statistically significant.

As regards the proportion of correct AS, a main effect of condition was found, *F*(2,44) = 3.96, *p* = 0.03, η*^2^_*P*_* = 0.15. Patients made more correct AS in overlap condition (*M* = 33.71, SD = 5.66) than step conditions (*M* = 24.33, SD = 22.10, *p* = 0.04). There was no difference between gap (*M* = 25.96, SD = 26.05) and overlap (*p* = 0.09) or step conditions (*p* = 0.89). ANOVA neither revealed main effect of patients [*F*(1,22) = 0.31, *p* = 0.58, η*^2^_*P*_* = 0.01] nor patients × conditions interaction [*F*(2,44) = 2.14, *p* = 0.13, η*^2^_*P*_* = 0.09].

##### Antisaccade Errors

As regards AS error latency, we found a main effect of conditions, *F*(1.38,30.29) = 36.47, *p* < 0.001, η*^2^_*P*_* = 0.62. Saccades were faster in gap condition (*M* = 249.38, SD = 42.40) than in step (*M* = 298.17, SD = 43.29, *p* = 0.004) and overlap conditions (*M* = 352.26, SD = 68.04, *p* < 0.001), and they were faster in step than overlap condition (*p* < 0.001). The main effect of patients [*F*(1,22) = 2.04, *p* = 0.17, η*^2^_*P*_* = 0.09] and the patients × conditions interaction [*F*(1.38,30.29) = 1.49, *p* = 0.24, η*^2^_*P*_* = 0.07] were not statistically significant.

The gap–step and the overlap–step ratios analysis showed a main effect of conditions [*F*(1,22) = 63.88, *p* < 0.001, η*^2^_*p*_* = 0.74]; gap–step ratio (*M* = −0.10, SD = 0.07) was lower than the overlap–step ratio (*M* = 0.10, SD = 0.12). Gap–step ratio and overlap–step ratio were statistically different from zero [*t*(23) = −7.34, *p* < 0.001, *t*(23) = 4.30, *p* < 0.001, respectively]. The main effect of patients [*F*(1,22) = 0.31, *p* = 0.59, η*^2^_*P*_* = 0.01] and patients × conditions interaction [*F*(1,22) = 0.85, *p* = 0.37, η*^2^_*P*_* = 0.04] were not statistically significant.

Analysis of variance on the proportion of corrected errors among the total of AS errors revealed only a main effect of patients [*F*(1,22) = 13.00, *p* = 0.002, η*^2^_*P*_* = 0.37]. Patients with SB made less corrections (*M* = 72.03, SD = 24.43) than patients with no SB (*M* = 95.13, SD = 7.95). The main effect of conditions [*F*(1.48,32.56) = 3.02, *p* = 0.08, η*^2^_*P*_* = 0.12], and the patients conditions interaction [*F*(1.48,32.56) = 2.35, *p* = 0.12, η*^2^_*P*_* = 0.10] did not reach statistical significance.

Finally, concerning the time to correct AS errors, we found a main effect of patients [*F*(2,44) = 8.99, *p* < 0.001, η*^2^_*P*_* = 0.29]. Patients with SB took more time to correct AS errors (*M* = 504.09, SD = 128.00) than patients with no SB (*M* = 384.82, SD = 95.43). A main effect of conditions was also found [*F*(1,22) = 9.46, *p* = 0.006, η*^2^_*P*_* = 0.30]. The time to correct AS errors was longer in overlap condition (*M* = 490.80, SD = 126.82) than in gap (*M* = 407.86, SD = 125.02, *p* < 0.001) and step conditions (*M* = 434.69, SD = 120.29, *p* = 0.02). There was no statistical difference between gap and step conditions (*p* = 0.56). Patients × conditions interaction did not reach statistical significance [*F*(1,22) = 0.02, *p* = 0.98, η*^2^_*P*_* = 0.001].

## Discussion

In this study, oculomotor impairments were found in elderly patients with SB. In AS tasks, although patients with SB and no SB had a similar proportion of correct AS, patients with SB had fewer corrected AS errors and they took more time to correct them than patients with no SB. Our results are consistent with the literature since Noiret et al. (2017) have reported a link between oculomotor impairments and inhibitory and cognitive flexibility measures in healthy elderly while other studies have found cognitive inflexibility among suicidal elderly (Neuringer, 1964; Richard-Devantoy et al., 2013). Our results suggested higher cognitive inflexibility in suicidal patients compared to non-suicidal. Cognitive flexibility can be defined as the ability to adapt cognitive processing strategies to face new and unexpected conditions in the environment (Cañas et al., 2003). Flexibility function impairment could explain the difficulty of depressed elderly with SB in generating solutions to the resurgence of SI to respond adequately to stressful environments as well as the difficulty to correct their errors in AS task.

Moreover, both groups had similar performances for PS latency. This result suggests that patients with SB do not result in a more pronounced decline in processing speed affecting saccade triggering than patients with no SB. Regardless of the groups, our study showed an increase in PS, correct AS and AS error latencies in overlap conditions compared to gap and step conditions. These results are in agreement with reported literature about gap and overlap effect (Saslow, 1967; Fischer and Boch, 1983; Kalesnykas and Hallett, 1987; Braun and Breitmeyer, 1988; Fischer et al., 1993; Crevits and Vandierendonck, 2005; Kristjansson, 2007; Noiret et al., 2017). Overlap condition requires attentional disengagement of the dot, which may explain higher saccadic latency than in gap and step conditions. Our results suggest a similar disengagement process and attentional capture for both patient groups in PS and AS tasks.

Some studies (Keilp et al., 2008, 2013) have shown that poorer performance on the Stroop task suggested a relatively independent marker of suicide risk within the context of depression. However, in our study, the neuropsychological assessment not only showed similar results between both groups in term of attention, processing speed, verbal episodic memory but also EF. These results support the idea that the SEM might be a more relevant tool for studying SB in the elderly. In our experiment, simple measures, such as reaction time (i.e., latency) or correct/incorrect trial, were failed to differentiate between patient groups. However, the precision and the continuous recording of the data during trials had produced finer variables (i.e., proportion of corrected errors and time to correct errors). These variables showed that correcting errors is more difficult for patients with SB than for patients with no SB.

A possible limitation of this study is related to the effect of drugs on SEMs. Some studies showed that drugs can affect eye movements (Wells et al., 2014), while others did not show any treatment effect on eye movements in depressed patients (Katsanis et al., 1997; Flechtner et al., 2002). To reduce the effects of the drugs, all of our patients took psychotropic drugs from the same family and were in a stable phase of their disease. Another limitation of this study was the small number of participants due to the difficulty to enroll this type of population. Furthermore, we used a convenience sample of inpatients that may not be representative of community-dwelling older adults. A larger sample size using randomly recruited representative samples should be needed to replicate our findings and further specifying SEM characteristics in patients with SB.

Our main findings showed that elderly depressed patients with SB had difficulties in correcting errors in AS task in comparison with elderly depressed patients with no SB. The assessment of eye movement parameters in depressed elderly patients with SB maybe a first step in identifying patients at risk. Our results are hopeful and encourage further research to confirm the interest in using eye movement measurements as a complementary tool for EF evaluation and to predict suicidal risks in the elderly patients.

## Data Availability Statement

The raw data supporting the conclusions of this article will be made available by the authors, without undue reservation.

## Ethics Statement

The studies involving human participants were reviewed and approved by the Committee for the Protection of Persons (CPP) and was conducted in accordance with declaration of Helsinki Declaration as revised in 1989. The patients/participants provided their written informed consent to participate in this study.

## Author Contributions

YB: study concept and design, methodology, analysis and interpretation of the data, preparation of the manuscript, and revising the manuscript. NN: study concept and design, statistical analysis and interpretation of the data, methodology, and revising the manuscript. BL: analysis interpretation and acquisition of the data. JM: funding acquisition and project administration. CD: acquisition of the data. JH: patient enrollment. CMa and DB: study concept and design. SR-D, CMo, EH, and EL: study concept and design and funding acquisition. PV: study concept and design, funding acquisition, project administration, patient enrollment, and revising the manuscript. GC: study concept and design, methodology, and revising the manuscript. All authors contributed to the article and approved the submitted version.

## Conflict of Interest

The authors declare that the research was conducted in the absence of any commercial or financial relationships that could be construed as a potential conflict of interest.

## Publisher’s Note

All claims expressed in this article are solely those of the authors and do not necessarily represent those of their affiliated organizations, or those of the publisher, the editors and the reviewers. Any product that may be evaluated in this article, or claim that may be made by its manufacturer, is not guaranteed or endorsed by the publisher.

## Abbreviations

AS: Antisaccade
AVF: Alternate Verbal Fluency
C-SSRS: Columbia Suicide Severity Rating Scale
C/W: Stroop color interference
EF: executive function
MMSE: Mini-Mental State Examination
PS: Prosaccade
PVF: Phonemic Verbal Fluency
SA: Suicide Attempt
SB: Suicidal Behaviors
SEM: saccadic eye movements
SI: Suicidal Ideation
SVF: Semantic Verbal Fluency
TMT: Trail Making Test.
